# Multiple reinforcement measures of flysch landslide

**DOI:** 10.1371/journal.pone.0290099

**Published:** 2023-08-24

**Authors:** Weidong Wang, Mingzhou Bai, Qinglin Liu, Haile Fitsume Fekadu

**Affiliations:** 1 School of Civil Engineering Beijing Jiaotong University, Beijing, China; 2 China First Highway Engineering Co. Ltd., Beijing, China; China University of Mining and Technology, CHINA

## Abstract

This work is mainly intended to investigate the flysch landslide reinforcement measures used in the Smokovac-Mateševo section of the North-South Expressway project in Montenegro. Bentley’s Plaxis software is used for a numerical analysis of sliding surface parameters of flysch strata in the limit equilibrium state. This study analyzes the slope safety factor for rreinforcement measures such as rock bolts, retaining walls, anti-sliding piles, slope unloading and bolt anchoring and obtains an optimal combination of reinforcement application for the flysch landslide. The effects of seismic action on complex stress and the discontinuous stress boundary conditions arising from various reinforcement measures on landslide stability are also examined. The measures applied in this paper can be used as a reference for flysch landslide reinforcement or other similar slope engineering measures.

## Introduction

Flysch, a sedimentary structure primarily composed of sandstone, shale, mudstone, and other rock masses, is predominantly found in semi-deep and deep-sea environments [1]. This type of formation, sometimes referred to as "sandwich" strata, exhibits distinct anisotropic mechanical properties due to the sequence of its thin layers which ultimately form larger rock masses [2–8]. Flysch is widely distributed around the world, notably in Eastern Europe, and has given rise to research interest on the stability of flysch slopes, particularly in relation to deforestation and weathering on low-undulating mountain structures [9–16].

**Fig 1:**
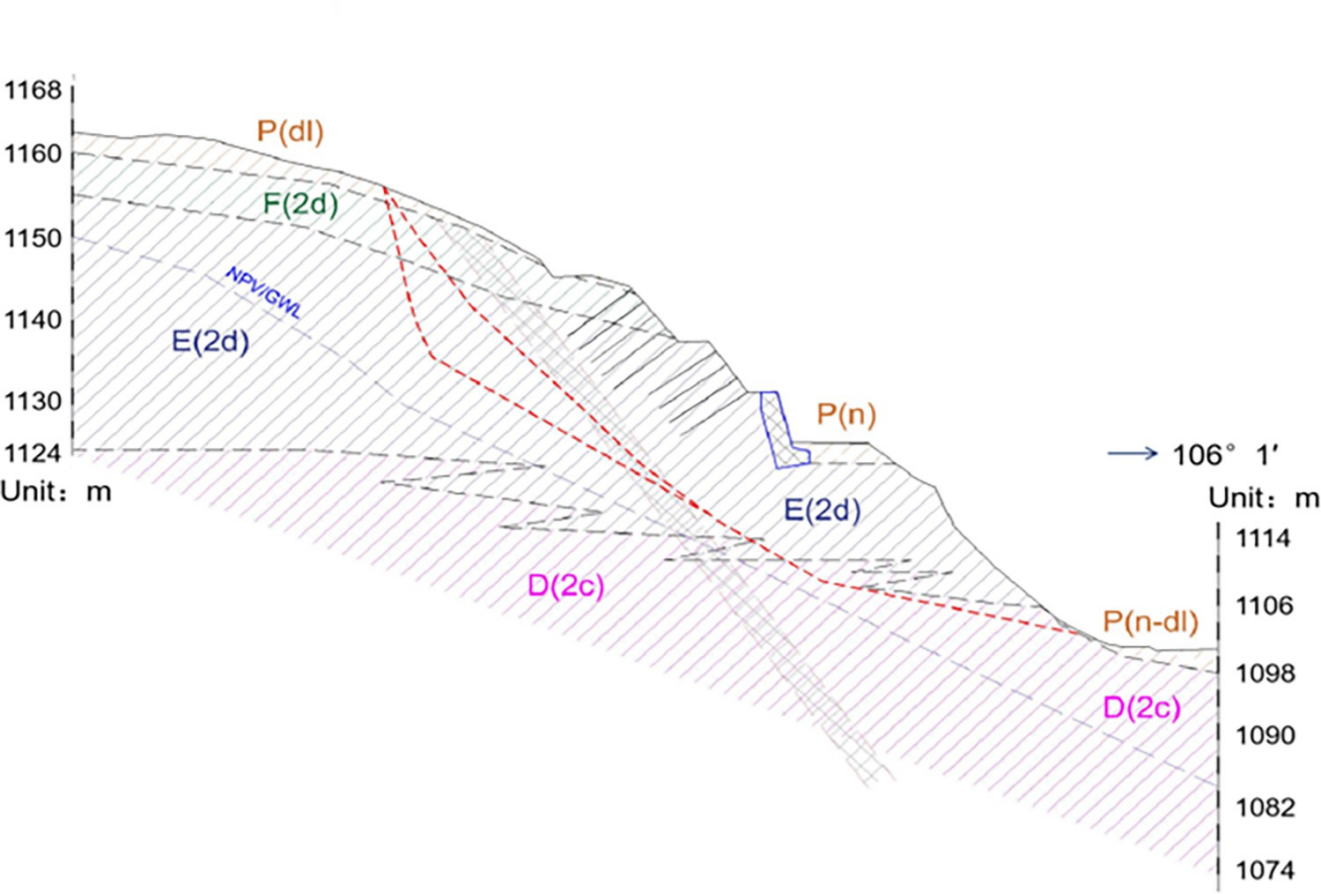
Schematic diagram of slope profile.

Our study focuses on the complex topography of the Smokovac-Mateševo section of the North-South Expressway construction project in Montenegro, where the flysch stratum is a dominant feature. Comprising of inter-bedded sandstone and thin layers of sandy marl and shale, the flysch formation is especially vulnerable to brittleness, weathering, and softening when exposed to water and air. The area is characterized by a high content of weak rock mass, particularly argillaceous rock along the highway, rendering the flysch formation relatively susceptible to degradation, failure, and in-situ landslides. The details on the region’s geological survey data, including the bulk weight, angle of internal friction, and cohesion, are presented in Table 1, while the rock structure on the slope is depicted in Fig 1, which provide insight into the physical and geometric characteristics of the soil and rock layers found in the location.

**Table 1:** Geological parameters for diluvium and flysch.

Material	Geotechnical category	Bulk weight	Angle of interior friction	Cohesion	Deformation modulus	Poisson’s ratio
		γ (kN /m ^3^)	ϕ (°)	c (kPa)	D (MPa)	n
**Diluvium**	P^a^	20	25	10	20	0.34
**Siltstone domination**	F(2d)	22.5	26	50	60	0.33
**Siltstone-sandstone**	E(2d)	23.5	29	320	800	0.32
**Siltstone-sandstone**	D(2c)	25	32	2500	2500	0.30

^a^ include P(dl): Diluvium, silt—clay material with fine debris, or locally with coarse debris; P(n): Rockfall material, blocks and debris with a silt-sandy properties; P(n-dl): Embankment in mixture with diluvium, mostly made of debris with silt-sandy properties.

Despite significant advances in geotechnical slope research, flysch slopes and their corresponding protection technologies remain inadequately studied. Some scholars have examined the effect of geological slope evolution and sliding mechanisms in large flysch formations [17–23], while others have concentrated on tunnel support technologies within flysch strata [24, 25]. Yang et al. [26] investigated the impact of different anchoring angles of bolts on flysch slope stability. The research on integrated treatments of flysch slopes, however, still leaves much to be desired.

The construction of roads in flysch regions presents landslide risks when the naturally occurring slopes are disrupted, necessitating comprehensive research into the treatment and protection of unstable flysch formations. The disruption of naturally occurring slopes poses landslide risks for the construction of roads in flysch regions, thus entailing comprehensive research into the treatment and protection of unstable flysch formations. Arbanas et al. [27] discussed reinforcement and excavation of flysch slopes and suggested the use of multi-layer sprayed concrete on reinforced grids for support measures on the Adriatic highway near Rijeka, Croatia. Qiu et al. [28, 29] conducted numerical simulation analyses of flysch slope stability and the influence of bolt anchor technology, although theirs was limited in scope to rock bolts and did not consider other reinforcement methods.

In response, this paper employs numerical simulation to investigate the stability of flysch slopes and the effect of various landslide reinforcement measures (e.g., prestressed anchor retaining walls, anti-sliding piles, mass unloading, and anchor bolts) on the slope safety factor. We used PLAXIS, a widely accepted numerical simulation software, to analyse the stress superposition and discontinuous boundary conditions brought about by seismic events and reinforcement support structures, as well as the subsequent changes in slope safety factors and their impact on slope stability. Given the limited research on the combined effects of flysch landslide reinforcement technologies, this study aspires to offer valuable data on the efficacy of slope protection measures for similar rock formations, in an attempt to provide a reference for future works.

## Methodology

### Basic conditions

**Fig 2:**
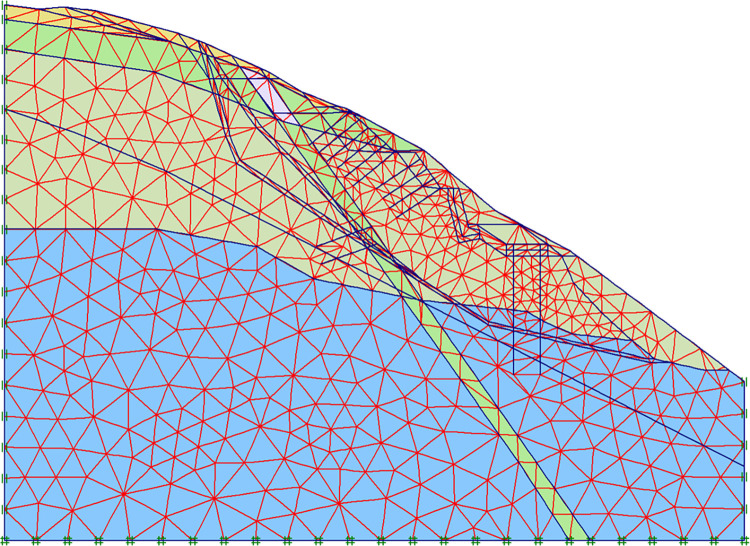
Numerical simulation network model.

The structural analysis in this paper was carried out using Plaxis software. Here, the calculation models comprised the following four types of elements: continuum elements, beam elements, geogrid elements and anchors, numerical simulation network model established is shown in Fig 2.

### Modeling

The model design and the calculation process is according to the following five steps: defining the soil materials, creating the geometry, applying the boundary conditions, generating the mesh and performing the analysis.

1. Defining the soil (supporting structures) materials: the parameters for the rock strata and supporting structures are defined using values shown in Tables 1 and 2, and the vertical and horizontal stresses at any location on the slope can be determined using the equations *σ*_*v*_ = *γ·h* and *σ*_*h*_ = *K*_*0*_*·σ*_*v*_ respectively (where *γ* is the unit weight of the soil, *h* is the depth below the ground surface, and *K*_*0*_ = 1—*sin(ϕ)*, with *ϕ* being the friction angle of the soil).
2. Creating the geometry: The slope’s dimensions, layers, and any other relevant features, such as retaining walls or anchors are defined and modeled.
3. Applying the boundary conditions: Since the flysch and anti-sliding pile have different stiffness values, it is essential to consider the boundary conditions between rock-soil mass and anti-sliding pile elements. For "Discontinuous boundary conditions," the element stress value at the contact point between the flysch boundary plane and anti-sliding pile should be equal so as to satisfy boundary conditions.
4. Generating the mesh: Automatic mesh generation tool is used to create a customized mesh to ensure that the mesh is fine enough to accurately capture the slope’s behaviour.
5. Performing the analysis: Construction stages are defined for the model, and the analysis is run.

**Table 2:** Structural parameters of the reinforcement unit.

	Cross section area	Moment of inertia	Modulus of elasticity	Axial stiffness	Bending stiffness	Poisson’s ratio
	A (cm^2^)	I (cm^4^)	E (Gpa)	EA/d(kN/m)	EI/d(kNm^2^/m)	n
**Gravity retaining wall**	-	-	31.5	6300000	2100000	0.15
**Anti-sliding pile**	17662.5	24837890.6	31.5	15904313	2236544	0.2
**Prestressed anchors**	6	-	195	117000	-	-

The seismicity of the system is considered in the "Inconsistent Boundary Conditions and Seismicity" section. The applied seismic loads correspond to the historic maximum of the project area. In all numerical simulations presented in this paper, slope instability is assumed to be reached when the Factor of Safety (FOS) approaches 1.

## Numerical simulation and results

### Result of back analysis

**Fig 3:**
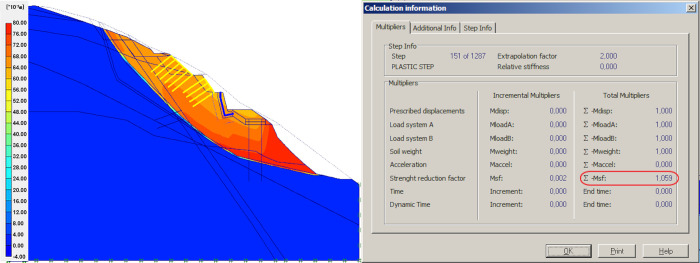
Back analysis method model and simulation results.

Since slope failure is determined by the strength of the potentially sliding plane, rather than the strength of the rock mass, we use back analysis to gradually reduce the applied sliding plane strength parameters to reach a Factor of Safety (FOS) near 1.0 (Fig 3), and to determine values leading to rock collapse under these conditions. The parameters reached in sliding plane leading to Factor of Safety (FOS) of 1.059 are: c = 1kPa and *ϕ* = 32.5°, which are the cohesion parameter and internal friction angle of the slope sliding surface, respectively. In the limit equilibrium state, numerical simulation method is used to analyse the relationship between reinforcement measures such as retaining walls, anti-sliding piles, anchor bolts and the safety factor of the slope. Sliding plane strength parameters determined in this step were used for all calculations in the following steps.

### Result of gravity retaining wall fixing

**Fig 4:**
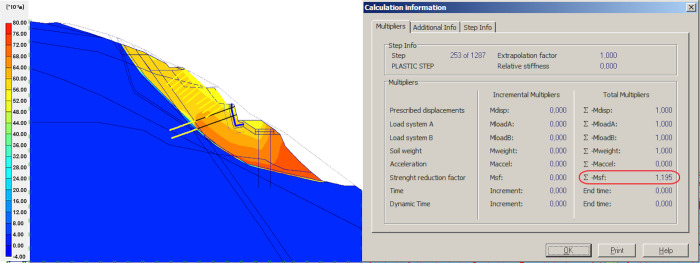
Prestressed anchor gravity retaining wall model and simulation results.

As the first measure of treatment, fixing of gravity retaining wall is introduced. The wall is fixed with prestressed anchors, anchored in RC frame cast on the surface and bonded to the rock category E, and partially to the rock behind the fault line. The model shows an increase in global stability. The slope FOS is shown in Fig 4, where the FOS increased to 1.195. Compared with the limit equilibrium state, the prestressed anchored retaining wall has raised the global stability by 0.136.

### Result of anti-sliding pile

**Fig 5:**
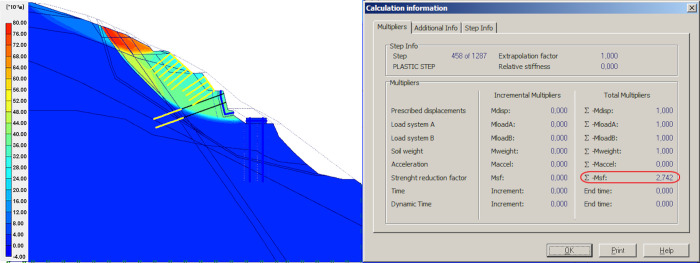
Anti-sliding pile model and simulation results.

After applying prestressed anchors on the retaining wall, two rows anti-sliding piles, 1.50 m in diameter, are constructed at the bottom of the slope. The model’s global stability is checked, as well as the contribution of the anti-sliding piles. As shown in Fig 5, the slope FOS increased significantly from 1.195 to 2.742, which shows that the anti-sliding pile is more effective than the retaining walls and other measures in improving the stability of flysch slope.

### Result of unloading

**Fig 6:**
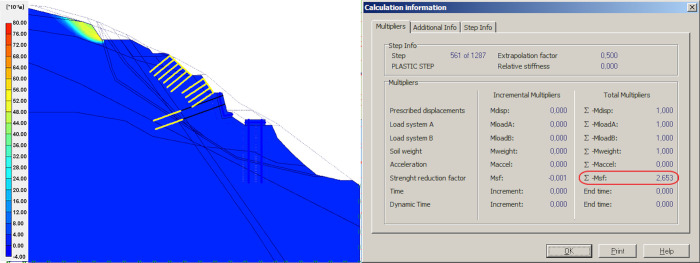
Results of the first unloading and simulation.

**Fig 7:**
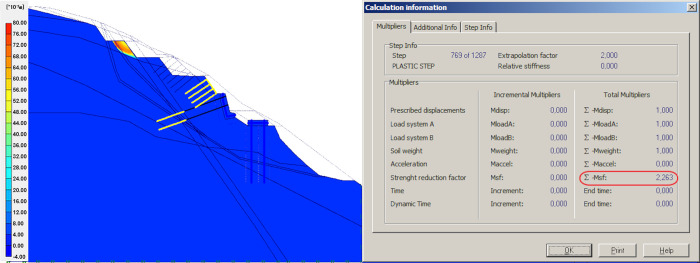
Results of the second unloading and simulation.

**Fig 8:**
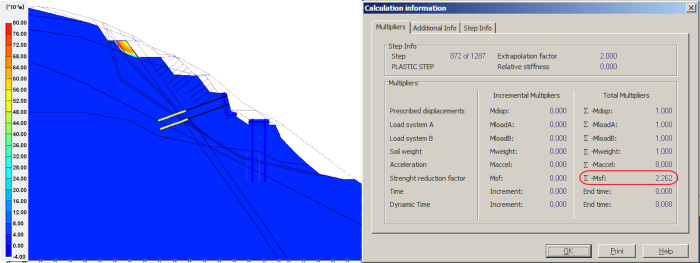
Results of third unloading and simulation.

**Fig 9:**
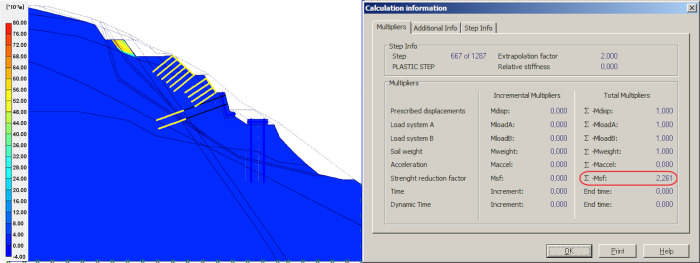
Results of the fourth unloading and simulation.

Unloading refers to the removal of the upper sliding body on top of the slope. This step involves dividing the upper portion into four parts, showing the changes on global FOS. For calculation, the effect of the anchors with steel mesh was neglected, the mesh structure was adopted for local protection of the slope above the retaining wall, and further, the mesh structure has no effect on global stability. The models and simulation results from the first to the fourth unloading of sliding body upper part are shown in Figs 6–9.

### Result of prestressed anchors

**Fig 10:**
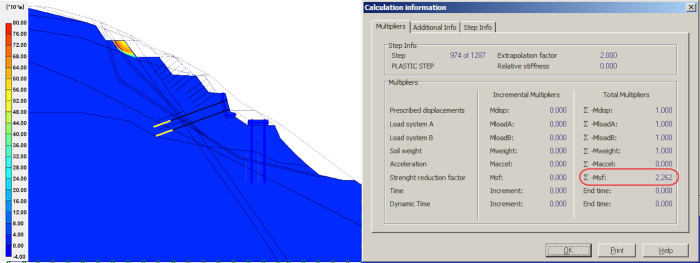
Prestressed anchor model simulation results.

The additional prestressed anchors with bonding part extending through the sliding body were introduced. Global FOS and sliding surface did not change from previous steps as shown in Fig 10. The prestressed bolt virtually has no anti-sliding protection for the flysch sliding body. As the prestressed bolt only connected the flysch layered structure, changing the rock and soil mass from its original plane stress state to three-dimensional state can prevent the internal sliding of the flysch. Prestressed anchors, therefore, did not reach the deep rock mass. Prestressed anchor bolts that were used to increase the anti-sliding resistance of the slope were not effective, and the FOS and the stability of the slope remain unchanged.

### Result of slope seismic stability

**Fig 11:**
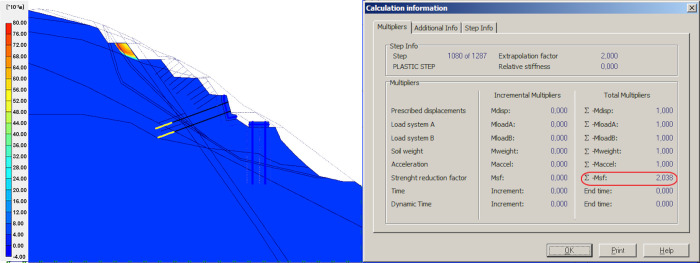
Result of slope seismic stability.

Seismic loading was then introduced based on the previous step for the permanent structure design. The design seismic forces F_H_ (horizontal, equal to 0.0706W) and F_V_ (vertical, equal to ± 0.33F_H_), acting on the ground mass are introduced. W is the weight of the sliding mass (defined in the software model with geometry, ground layers and structural unit weights). The seismic wave is bound to decay when propagating through the heterogeneous flysch layers, but the maximum distance of the flysch layers is relatively small (<50m). The attenuation and reduction of seismic waves is, therefore, not considered in the numerical simulation. Calculation shows that seismicity exhibits a local effect after the introduction of the seismic loads. However, global FOS still stands at a high level of 2.038, as shown in Fig 11. Here, if seismic wave attenuation is considered, the safety factor will be even higher, indicating that the combined measures used to prevent flysch slope instability are effective under seismicity.

### Discontinuous boundary conditions

According to the assumption of the above theoretical slope model, the displacement and deformation between the reinforcement structure and the rock and soil mass are discontinuous. The anti-sliding piles are the most effective protection measure. There is a discontinuous boundary of displacement and deformation between the reinforcement and surrounding materials. In this step, the numerical simulation analysis will consider the effect of discontinuous boundary conditions between the anti-sliding pile and the rock and soil mass on the safety factor of the slope. This analysis follows the step where the new anchor bolts are applied.

**Fig 12:**
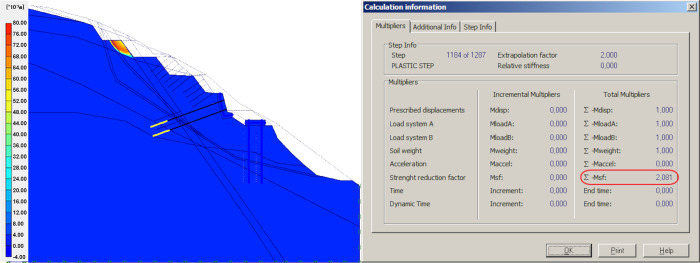
Result of introducing “inconsistent” boundary conditions.

In the numerical analysis, discontinuous boundary conditions are modelled by setting a discontinuous interface between the anti-sliding pile and the rock-soil mass in front of the pile. The stress states of the contacting surfaces of the anti-sliding pile and the rock-soil mass are equal on this discontinuous surface. The safety factor of the slope model is calculated, as shown in Fig 12.

The introduction of the described changes in the model reduced the global FOS from 2.262 to 2.081, but the critical sliding surface did not show any change because it is more affected by the local properties. Fu [30] used the modified finite element analysis method of anti-sliding pile and rock-soil mass interaction to conduct multiple numerical simulations, but without considering the discontinuous boundary conditions.

The stiffness equation of the rock mass is “condensed” on to the stiffness equation of the pile, and the resulting displacement of the rock mass is smaller than when the discontinuous boundary is considered. Moreover, the numerical analysis of this step considers the discontinuous boundary conditions of displacement and deformation between the rock-soil mass and the anti-sliding pile. The calculation shows the displacement is large, which caused a decline in slope stability and the slope’s FOS.

### Inconsistent boundary conditions and seismicity

**Fig 13:**
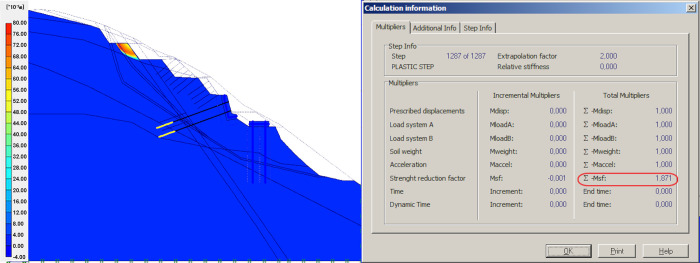
Result of inconsistent boundary conditions and seismicity.

In this step, seismicity is introduced on top of the previous step for permanent structure design case. The magnitude of earthquake inertia force (F_H_ and F_V_) is consistent with the "result of slope seismic stability" mentioned earlier. Here, the complex stress changes and discontinuous stress boundary conditions are investigated for slope reinforcement. As shown in Fig 13, when seismic loads are applied, the FOS decreased from 2.081 to 1.871, because the rain erosion and weathering increased the flysch soil’s freedom. But the critical sliding surface remained intact. Hence, the slope is very stable, further indicating that the slope support measures in this paper are highly effective for slope stability.

## Discussion

Numerical simulation method is used to analyze series of landslide treatment measures, and the safety factor and stability of the slope under seismic load and discontinuous boundary conditions are considered. The following is a comparative analysis of these landslide treatment measures, and their treatment effects are studied under seismic load and discontinuous boundary conditions.

**Fig 14:**
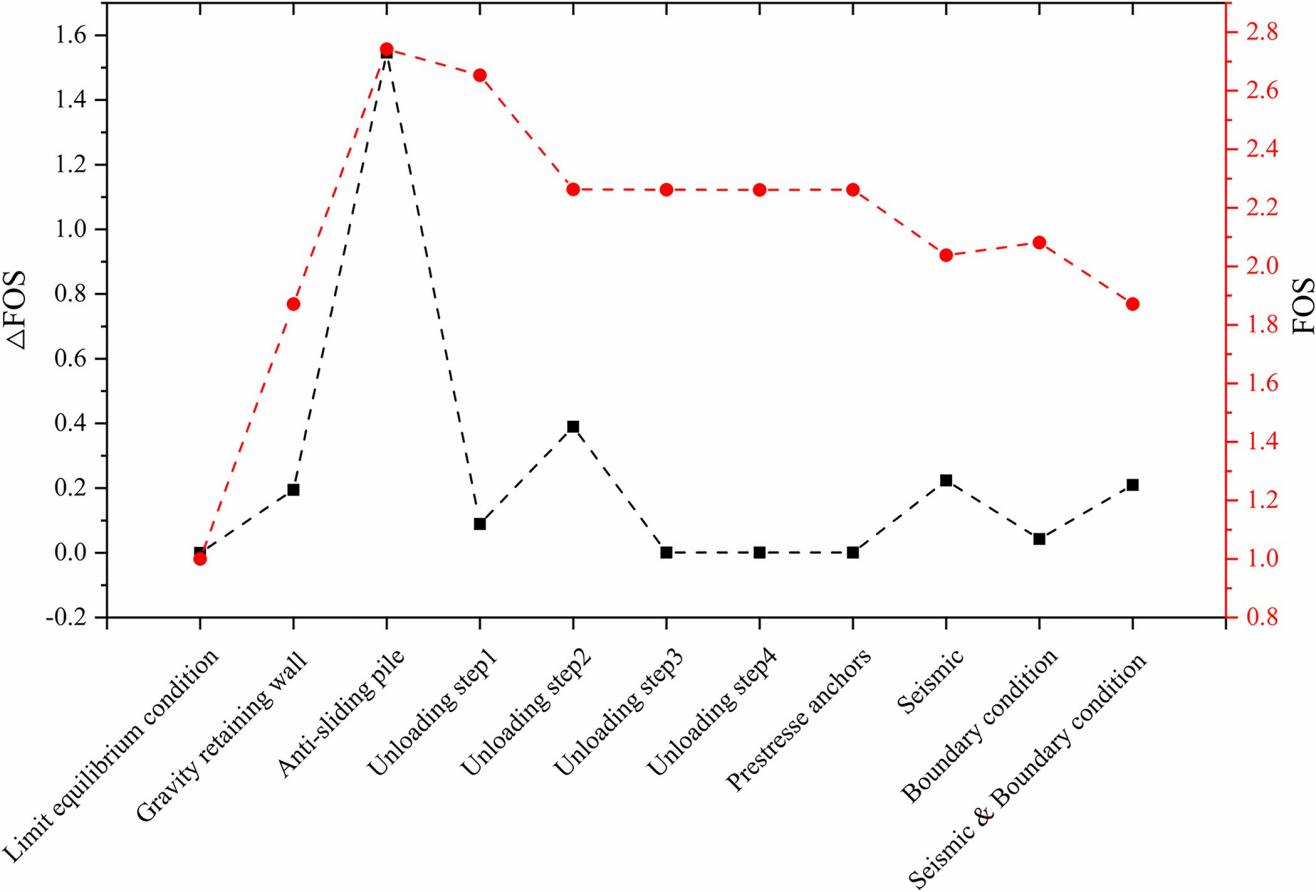
Variation of safety factor with different slope treatment measures.

As shown in Fig 14, in the numerical simulation analysis, FOS represents the calculated safety factor of the slope, and △FOS represents the change value of the calculated safety factor of the adjacent simulation test.

As can be seen from Fig 14, the anti-sliding pile measure shows the largest safety factor, 2.742. Compared with that of gravity retaining wall, the safety factor increased by 1.547, the largest change in safety factor in all simulation tests. Therefore, anti-sliding pile is the most effective singular measure for the reinforcement of flysch slopes, which can fast anchor the slope. This research result is consistent with study by Jiang [31] and Zhang [32], namely, anti-sliding pile is a dynamic and effective support measure for unstable slope.

In the four times of slope cutting and unloading at the upper part of the slope, the safety factor of the second unloading is reduced by 0.39 compared with the first unloading, which is mainly caused by the reduction of the upper sliding surface and the reduction of anti-sliding force provided by the upper flitting slope unloading. When the upper slope is cut and unloaded from the second time to the fourth time, the safety factor virtually shows no change, indicating that the effect of multiple unloading on improving the stability of the slope on the decrease, which coincides with Cavounidis’s [33] conclusion that load reduction in the upper and middle parts of the slope is more conducive to maintaining the stability of the slope.

The safety factor does not change visibly when the slope is reinforced by the bonded bolt, which indicates that the rock bolt does not produce obvious effect. Considering the boundary conditions between anti-sliding pile and rock mass, seismic load, the slope safety factor is reduced to a certain extent, but the slope safety factor remains large, indicating the slope is stable overall. Anti-sliding pile, gravity retaining wall and slope unloading measures are effective in treating unstable slope.

When considering the effect of earthquakes, the internal disturbance of the slope leads to the increase of instability, thus decreasing the safety factor, and the slope is prone to landslide. When considering the discontinuous boundary between anti-sliding pile and loose rock of flysch, the stiffness of loose rock of flysch is smaller than that of the concrete anti-sliding pile, and the deformation and displacement of rock and soil mass is larger than that of the pile. Therefore, when discontinuous boundary conditions between the flysch and pile are considered, the deformation and displacement of rock and soil mass are larger than those without consideration, so the slope stability decreases, that is, the safety factor decreases [29].

When a variety of landslide control measures are used, the order of implementation of the control measures has a great influence on the slope stability. In the treatment of the unstable flysch slope, the first consideration is to strengthen the gravity retaining wall, anchor the lower slope foot of the unstable slope, improve the unstable condition, and maintain the overall stability of the slope. Then take the most effective measures, anti-sliding pile to provide the main anti-sliding force support to ensure the overall stability. Then consider unloading of the upper parts of the slope, then anchor reinforcements to improve the local stability of the slope. After the above treatments, the slope stability under seismic load and discontinuous boundary conditions is tested. The implementation sequence of such control measures and numerical simulation sequence are suitable for such cases, which can provide technical and research references for similar projects.

## Conclusions

From the above steps of numerical analysis, we draw the following conclusions:

1. A series of combined measures such as anti-sliding piles, prestressed bolt retaining walls, sliding mass unloading, and prestressed bolts can increase flysch stratum’s slope stability and prevent landslides. The numerical simulation analysis results show that the anti-sliding piles at the lower part of the slope are the most effective measures.
2. The flysch slope is affected by the disturbance during excavation. After reinforcement measures are taken, the analysis of the slope stability under seismic action for the duration of the whole operational period of the highway shows the safety factor is still within a safe range, indicating that the reinforcement measures used for flysch landslide are reliable.
3. This flysch landslide reinforcement study adopts the combined use of various methods including prestressed anchor retaining wall, anti-sliding pile, sliding mass unloading, and bolt anchor reinforcement measures. This results in complex stress changes and discontinuous boundary conditions of the slope, which have a negative impact on the safety factor and stability of the slope. Therefore, the order of implementation of landslide reinforcement measures should be reasonably considered.
4. Regarding the flysch landslide, the upper and middle parts unloaded, and it is found that unloading the upper part is more effective than unloading the middle part in improving the safety factor and stability of the slope. When the unloading is performed in multiple steps, the improvement of the safety factor and stability decreases. Therefore, the unloading steps should be limited to a certain number.
5. Rock bolt cannot be used as anti-sliding anchor on flysch because its function is to change the plane stress state to three-dimensional stress state. This can prevent the dislocation between flysch rock layers, but is ill-suited for increasing the sliding mass’s global stability.
6. Discontinuity in stress boundary has an impact on the stability of slopes. Therefore, when various reinforcement measures are taken for the unstable slopes, the reasonable and scientific implementation of support and reinforcement measures becomes crucial. Effective management of slopes will also affect their stability.

The above research shows that anti-sliding piles are the most effective singular type of measures to prevent landslides. However, instead of merely using anti-sliding piles or if conditions do not permit the use of piles, recommended order of implementation of these measures is imperative. First, gravity retaining walls should be used to prevent landslides on unstable slopes and reduce the damage of the slope structure. Then, anti-sliding piles should be used at the lower part of the slope, so that the gravity retaining wall can act as an anchor to the sliding body (to prevent sliding). Slopes generally slide during the construction of anti-sliding piles at the lower part of the slope and the compression of the pile finally comes into effect to stop further sliding. If anti-sliding piles are adopted first, the unstable slope will not have the support of anti-sliding piles’ anchoring force. The disturbance from construction at the lower part of the slope will then cause serious landslide of the slope. The order of reinforcement measures in this paper can be used as reference in similar projects in the future.
